# Radiotherapy-Related Fatigue Associated Impairments in Lung Cancer Survivors during COVID-19 Voluntary Isolation

**DOI:** 10.3390/healthcare10030448

**Published:** 2022-02-26

**Authors:** Alejandro Heredia-Ciuró, Isabel Castillo-Pérez, Antonio Lazo-Prados, María Granados-Santiago, Laura López-López, Araceli Ortiz-Rubio, Marie Carmen Valenza

**Affiliations:** 1 Department of Physiotherapy, Faculty of Health Sciences, University of Granada, 18016 Granada, Spain; ahc@ugr.es (A.H.-C.); mariagranados@ugr.es (M.G.-S.); aortiz@ugr.es (A.O.-R.); cvalenza@ugr.es (M.C.V.); 2 Oncological Radiotherapy Service of the “Hospital PTS”, Clínico San Cecilio University Hospital, 18016 Granada, Spain; isabel.castillo.sspa@juntadeandalucia.es (I.C.-P.); lll92hs@hotmail.com (A.L.-P.)

**Keywords:** lung cancer, COVID-19 lockdown, radiotherapy, fatigue, impairments

## Abstract

The main objective of this study was to investigate the impairments presented after COVID-19 voluntary isolation by lung cancer survivors that experienced radiotherapy-related fatigue. In this observational study, data were collected after COVID-19 voluntary isolation. Patients were divided into two groups according to their fatigue severity reported with the Fatigue Severity Scale. Health status was assessed by the EuroQol-5D, anxiety and depression by the Hospital Anxiety and Depression Scale, and disability by the World Health Organization Disability Assessment Schedule 2.0. A total of 120 patients were included in the study. Patients with severe fatigue obtained higher impairment results compared to patients without severe fatigue, with significant differences in all the variables (*p* < 0.05). Lung cancer survivors who experienced severe radiotherapy-related fatigue presented higher impairments after COVID-19 voluntary isolation than lung cancer patients who did not experience severe radiotherapy-related fatigue, and showed high levels of anxiety, depression and disability, and a poor self-perceived health status.

## 1. Introduction

Concurrent chemo-radiation remains the standard treatment for most cancer patients [1]. Radiotherapy is an integral part of the multidisciplinary treatment of thorax and lung cancer [2], being indicated before and after surgery [3], after chemotherapy in unresectable tumors staged as extensive disease [4], and for frail patients for whom surgery is not recommended [5].

Radiotherapy is an oncological treatment that implies the apoptosis of both tumoral cells [6] and normal cells due to radiation toxicity [7], resulting several side effects. The side effects of radiotherapy are an important factor that explains to a large extent the poor survival compared to surgery [8]; these treatments can lead to musculoskeletal and neuromuscular complications, or the dysfunction of a visceral organ such as the heart or the lungs [9]. Among all side effects (pain, cough, dispnoea, insomnia, oesophagitis, weight loss, nausea, erythema [10]), the fatigue is one of the most common symptoms reported [11,12]. Jones et al. 2016 concluded that one third of cancer survivors suffer clinically relevant levels of fatigue up to 6 years post-radiotherapy treatment [13].

Cancer-related fatigue (CRF) damages the quality of life of cancer patients [14]. Tt interferes with their daily activities [15], is associated with high levels of disability [13], and is reported as highly distressing. However, despite the adverse impact and the high prevalence, health care practitioners infrequently address it, and its impact on the quality of life of cancer patients is underestimated [16]. CRF is multifactorial; it is probably related to psychological and biochemical disorders [17], in addition to several negative health outcomes to be taken into account when managing it, including post-exertional malaise [18], physical pain, unrefreshed sleep [19], and poor general health status [20]. Furthermore, attention must be paid to the development of anxiety, depression, and other co-occurring physical symptoms as contributing factors [13]. A recent review demonstrated that CRF is reduced by exercise [21], but in the same way, reduced activity levels increase fatigue, and further reduce functional capacity and quality of life [22], even showing concerned effects on survivorship [23].

The COVID-19 pandemic has impacted lives around the world, causing high physical and psychological suffering, such that 40% of lung cancer patients have had their quality of life affected by home confinement [24]. The physical activity levels of cancer survivors have declined due to the lack of physical exercise and the mentioned suffering [25,26]. Moreover, the lockdown triggered by COVID-19 has led to an increase of distress among Spanish cancer patients [27], disrupting their psychological well-being and favoring the development of psychiatric disorders in these patients [28]. With all of the above, published studies have found that many people have an increased perception of fatigue during the COVID-19 era [29,30], highlighting possible psychologically-related symptoms of lockdown, quarantine, social distancing, and unprecedented pressure in daily life.

Considering the scientific background that relates perceived fatigue and presented disability with voluntary isolation, and the lack of awareness of the impact of the COVID-19 lockdown on lung cancer patients, the purpose of this study was to investigate the impairment presented after COVID-19 voluntary isolation in lung cancer survivors that experienced radiotherapy-related fatigue.

We hypothesized that lung cancer patients who experienced severe radiotherapy-related fatigue present higher impairments during COVID-19 voluntary isolation than lung cancer patients who did not experience severe radiotherapy-related fatigue.

## 2. Materials and Methods

### 2.1. Participants and Study Design

A cross-sectional observational study was performed. Patients were recruited from the Oncological Radiotherapy Service of the “Hospital Universitario San Cecilio” (Granada, Spain), between June 2020 and May 2021. The included lung cancer survivors were aged 18–80 years, treated by radiotherapy treatment, and all were informed and signed the informed consent. Exclusion criteria were a diagnosis of fibromyalgia or similar condition, diagnosis of any psychiatric disorder, diagnosis of COVID-19 in the previous year, being in actual chemotherapy treatment, and any cognitive impairment affecting the possible completion of the evaluation protocol. We conducted this study in accordance with the Declaration of Helsinki 1975, revised in 2013. The study protocol was reviewed and approved by the Biomedical Research Ethics Committee of Granada (Granada, Spain). 

### 2.2. Group Assignment

Patients were divided into two groups—a group with severe fatigue and another group without severe fatigue—according to the cut-off point of the Fatigue Severity Scale (FSS), which was recorded after COVID-19 voluntary isolation. The FSS has been used in different chronic conditions [31,32,33], including advanced cancer [34]. This scale includes nine items that are scored on a seven-point scale, ranging between 9 (minimum fatigue) and 63 (maximum fatigue). Cancer patients with a scores of 42 or greater were included in the group with severe fatigue, and cancer patients with scores lower than 42 were included in the group without severe fatigue [35]. This cut-off point has been proven in previous studies of CRF [34].

The FSS has been used in previous cancer populations in more than 400 occasions [36]. It has shown a good internal consistency in cancer subjects (Cronbach’s α = 0.96) and in healthy subjects (Cronbach’s α = 0.88). Additionally, it has a good correlation with the European Organization for Research and Treatment of Cancer (EORTC) Fatigue Scale (Rs = 0.83) and the bidimensional fatigue scale (Rs = 0.62), demonstrating its validity as a measure of fatigue.

### 2.3. Outcome Measures

Data were recorded after COVID-19 voluntary isolation. Anthropometric data, characteristics of the pathology, adjuvant treatment, and characteristics of radiotherapy treatment [37] were collected from medical history at admission.

The main study outcomes evaluated the patient affectation including anxiety and depression levels, disability, and self-perceived health status.

The Hospital Anxiety and Depression Scale (HADS) was used to assess anxiety and depression. The HADS is a self-reported measure that contains 14 statements ranging from 0 to 3, which result in two subscales: anxiety (0–21) and depression (0–21). A score of 8 is an indicator of possible anxiety or depression [38]. This scale has presented good reliability and validity in Spanish populations [39], and it has been used in cancer patients [40]. 

Disability was measured by the World Health Organization Disability Assessment Schedule 2.0 (WHODAS 2.0). WHODAS 2.0 has good validity and high reliability [41,42]. This scale observes how patients are able to perform their activities. It contains six domains divided into 36 items, scoring from 1 (slight) to 5 (extreme/unable to do it); the total score ranges from 36 to 180, where greater scores mean greater disability [43].

The EuroQol-5D was used to assess self-perceived health status [44]. It has five items that evaluate five dimensions of health status: mobility, self-care, usual activities, pain, and anxiety and depression. Additionally, it has a visual analog scale ranging from 0 (the worst imaginable health) to 100 (the best imaginable health), where the patients indicate their self-perceived health status. The Spanish version of the EuroQol-5D has good validity and high reliability [44]. 

### 2.4. Statistical Analysis

IBM SPSS version 23.0 was used to perform the statistical analysis [45]. The Kolmogorov–Smirnov test was used to assess the normal distribution of the data, while Fisher’s F-test determined the homogeneity of variances. Numerical variables were expressed as mean ± SD. When both conditions were achieved, a parametric test (Student’s *t* test) was used; when any conditions were not achieved, a nonparametric test (Mann–Whitney test) was used. In any cases, α = 5%. Previous to the between group comparison, two groups were created according to fatigue status: having (≥42 FSS score) or not having (<42 FSS score) severe fatigue. 

## 3. Results

A sample of 130 patients lung cancer survivors treated with radiotherapy was screened in this study. From that sample, 10 patients were excluded due to difficulties communicating with the interviewers (n = 6) or not accomplishing the voluntary isolation (n = 4). Finally, 120 were included in the study. The 120 participants gave their consent to be evaluated and all completed the evaluation. When the presence of fatigue was evaluated, 80 patients did not present severe fatigue and 40 participants presented severe fatigue. 

The characteristics of the participants are summarized in Table 1. Of the 120 lung cancer survivors enrolled, 80% were males and 20% were females. The study sample had an average age of 64.1 and 65.3 years in the groups. The cancer entity of the patients without severe fatigue was non-small cell lung cancer. In the group with severe fatigue, 20 participants presented non-small cell lung cancer, and the other 20 patients presented small cell lung cancer. Of all patients, 33.3% received surgery and 66.6% received chemotherapy. The number of radiotherapy sessions had a heterogeneous distribution that was different between the groups.

The comparison of the main study outcomes between groups, using Student’s *t* test and the Mann–Whitney test, are presented in Table 2. The HADS results present significant differences between groups (*p* < 0.001), with higher results in the group with severe fatigue.

All the domains of the presented disability questioner also had significant differences (*p* < 0.001), presenting worse results in the group with severe fatigue for each domain and the total score.

The group with severe fatigue presented worse results in the Euroqol-5D scale, with significant differences in the mobility (*p* = 0.003), self-care (*p* = 0.036), activities of daily living, pain and anxiety, and depression (*p* < 0.001) subscores.

## 4. Discussion

The study aimed to identify impairments related to radiotherapy-related fatigue during COVID-19 isolation. As hypothesized, severe fatigue was associated with higher disability and worse anxiety and depression and perceived health status. 

CRF is defined by the National Comprehensive Cancer Network (NCCN) as “a distressing persistent, subjective sense of physical, emotional and/or cognitive tiredness related to cancer or cancer treatment that is not proportional to recent activity and interferes with usual functioning” [46]. Although voluntary isolation is a necessary action to reduce the spread of the virus, it can trigger changes in living habits that can represent a physiological challenge that can further interfere with usual functioning, implying significant health risks [47].

The high prevalence of radiotherapy-related severe fatigue found in this study is similar to that found in other studies, such as Tombal et al. [48]

Our study showed that lung cancer survivors who experienced severe fatigue presented higher level of anxiety and depression than those who did not experience severe fatigue. Our results are in line with previous studies [13] that reported a significant correlation between mood disturbances and experiencing significant CRF, finding high levels of depression in 67% of participants who experienced significant CRF, compared to 14% who did not experience CRF. However, our study is the first to study radiotherapy-related fatigue of lung cancer survivors in relation to COVID-19 voluntary isolation. 

The studies previous to the COVID-19 era [13] have investigated other cancer entities, finding, as in our results, that cancer survivors with significant CRF also presented high levels of disability and depression. Disability levels have great importance during voluntary isolations, considering that high levels of disability can make it difficult to perform the activities of daily life at home. In this way, because of the constraints that it produces, disability is now considered as important as mortality from the public health point of view [43].

With respect to the Euroqol-5D results, our study found significant differences between groups. A recent study of Presley CJ et al. [49], which studied lung cancer survivors with advanced stages, found alterations in 37.6% of the patients for usual activities, 26.6% for mobility, and 5.2% for self-care. Additionally, they also concluded that these results were significantly associated with psychological symptoms.

Our study results explore a possible effect of COVID-19 isolation on lung cancer survivors. Studies in healthy populations [29,30] have highlighted the presence of fatigue during COVID lockdown and the relation between social distancing and high pressure in daily life. Nevertheless, this has not yet been studied in cancer survivors, even though it has been demonstrated that cancer patients have severe stress symptoms and psychological distress. Particularly, those with lung cancer are at higher risk and may need special attention [50].

The stressors (physical, mental, emotional, financial, etc.) are directly related with the development of symptoms, given the high comorbid nature of mood disorders in patients with fatigue [51]. It has been concluded that the COVID-19 pandemic has disturbed the mood and, therefore, the fatigue of several patients [51]. In this way, in line with our results, Wang Y. et al. [52] observed a population of 6213 cancer patients during the COVID-19 pandemic where 23.4% presented depression, 17.7% had anxiety, and 13.5% had hostility; however, they did not observe the relation between these symptoms and the disability presented, as we did in this investigation.

Future studies might focus on psychological disorders and the presented disability. A shared-decision care plan could improve the recovery of these disorders, helping cancer patients to improve and deal with impairments related to radiotherapy treatment, especially lung cancer survivors who present severe fatigue. Guidelines on detection and treatment of CRF during active treatment, follow-up, and at end-of-life [53] have been developed by the American Society of Clinical Oncology and the Canadian Association of Psychosocial Oncology [54].

## 5. Limitations

This study presents several strengths, such as the high significance of the results, a high response rate, and the use of a validated and used CRF tool with a cut-off point that has good agreement with the current CRF diagnostic criteria [36]. However, the results must be interpreted taking into account the study limitations. A cross-sectional design was utilized, which provided a one-time estimate of CRF prevalence without follow-up over time, limiting our knowledge of the course of CRF. Additionally, the absence of a control group without isolation affects our ability to draw conclusions about the causality of associated factors.

## 6. Conclusions

Lung cancer survivors who experience severe radiotherapy-related fatigue present higher impairments after COVID-19 voluntary isolation than lung cancer patients who do not experience severe radiotherapy-related fatigue, show higher levels of anxiety, depression and disability, and have a poorer self-perceived health status.

An important finding of this study is that presented severe fatigue and the high psychological suffering of lung cancer patients are associated during COVID-19 voluntary isolation. 

Clinicians can use the findings of this study to identify lung cancer survivors who have a higher risk for developing greater radiotherapy-related impairments during voluntary isolation, permitting the early initiation of management interventions. In this line, therapeutic approaches need to, first, screen for fatigue in lung cancer patients routinely, and second, propose therapeutic intervention including exercise, psychological assessment, and functional training.

## Figures and Tables

**Table 1:** Clinical characteristics of the included patients.

Variable		Participants without Severe Fatigue (n = 80)	Participants with Severe Fatigue (n = 40)	Total (n = 120)	*p*
**Age, y**		64.1 ± 8.556	65.3 ± 9.381		0.485
**Sex**	Male	64 (80)	32 (80)	96 (80)	1
	Female	16 (20)	8 (20)	24 (20)	
**Cancer Entity**	NSCLC	80 (100)	20 (50)	100 (83.3)	0.015
	SCLC	0	20 (50)	20 (16.6)	
**Adjuvant Treatment**	Surgery	20 (25)	20 (50)	40 (33.3)	0.017
	Chemotherapy	60 (75)	20 (50)	80 (66.6)	
**Radiotherapy Sessions (Days)**	<33	27 (33.7)	13 (32.5)	40 (33.3)	0.265
	>33	53 (66.3)	27 (67.5)	80 (66.6)	

y: years; NSCLC: non-small cell lung cancer; SCLC: small cell lung cancer; Data are expressed as n (%) or mean ± SD.

**Table 2:** Primary variables compared between groups.

Variable	Patients without Severe Fatigue (n = 80)	Patients with Severe Fatigue (n = 40)	*p*
**HADS**			
Anxiety subscore	3.375 ± 4.589	9.75 ± 7.683	<0.001 **
Depression subscore	1.938 ± 2.06	8.5 ± 5.47	<0.001 **
HADS total score	5.313 ± 5.585	18.25 ± 11.022	<0.001 **
**WHODAS 2.0**			
Cognition subscore	7.125 ± 3.204	11.375 ± 5.469	<0.001 **
Mobility subscore	6.063 ± 2.376	12.75 ± 5.248	<0.001 **
Self-care subscore	4.313 ± 1.735	7.375 ± 3.799	<0.001 **
Relations subscore	5.5 ± 2.108	8.5 ± 3.767	<0.001 **
Housework subscore	6.75 ± 3.157	14.375 ± 5.746	<0.001 **
Work subscore	4.188 ± 4.174	11.5 ± 5.86	<0.001 **
Participation subscore	11.188 ± 4.676	18.875 ± 7.083	<0.001 **
WHODAS Total Score	45.25 ± 18.16	81.87 ± 26.26	<0.001 **
**Euroqol-5D**			
Mobility subscore	1.2 ± 0.513	1.5 ± 0.506	0.003 *
Self-care subscore	1.15 ± 0.658	1.4 ± 0.496	0.036 *
Activities daily life subscore	1.25 ± 0.539	1.7 ± 0.648	<0.001 **
Pain subscore	1.3 ± 0.56	2.1 ± 0.955	<0.001 **
Anxiety-depression subscore	1.35 ± 0.658	1.8 ± 0.607	<0.001 **
VAS subscore (0–100)	69.25 ± 20.268	62 ± 21.626	0.073

HADS: Hospital Anxiety and Depression Scale; VAS: Visual Analog Scale; WHODAS 2.0: World Health Organizations Disability Assessment Schedule 2.0; * *p* < 0.05; ** *p* < 0.001; Data expressed as Mean ± SD.

## Data Availability

The data presented in this study are available on request from the corresponding author.
